# Rice residue management alternatives in rice–wheat cropping system: impact on wheat productivity, soil organic carbon, water and microbial dynamics

**DOI:** 10.1038/s41598-024-52319-6

**Published:** 2024-01-21

**Authors:** Santosh Korav, Dharam Bir Yadav, Ashok Yadav, G. A. Rajanna, Jagdish Parshad, Sridevi Tallapragada, Hosam O. Elansary, Eman A. Mahmoud

**Affiliations:** 1 Choudhary Charan Singh Haryana Agricultural University, Hisar, Haryana 125004 India; 2 Department of Agronomy, School of Agriculture, Lovely Professional University (Phagwara) Jalandhar, Punjab, 144411 India; 3 ICAR-Directorate of Groundnut Research, Junagadh, Gujrat, 362001 India; 4 Plant Production Department, College of Food and Agriculture Sciences, King Saud University, P.O. Box 2460, 11451 Riyadh, Saudi Arabia; 5 Department of Food Science, Faculty of Agriculture, Damietta University, Damietta, 34511 Egypt

**Keywords:** Microbiology, Plant sciences

## Abstract

In the Indo-Gangetic Plains (IGP), rice–wheat cropping system (RWCS) predominates, producing large quantity of crop residue and its management is major concern. Farmers usually burn the residue to clear the field for succeding crop, and burning damages soil microbes, resulted in loss of soil organic matter. Hence, current study was conducted to assess the impact of different Happy seeder based residue management options on changes in microbial dynamics, enzyme activities and soil organic matter content and also to know that alternative method for attaining sustainable wheat productivity in sandy loam soils of Haryana, India. Results revealed that Zero tillage wheat (ZTW) with partial and full residue retention treatments sown with Happy seeder (after using chopper and spreader), and ZTW with anchored stubbles significantly enhanced soil microbial count by 47.9–60.4%, diazotropic count by 59.0–73.1% and actinomycetes count by 47.3–55.2%, grain yield by 9.8–11.3% and biomass yield by 7.4–9.6% over conventional tilled (CT) residue burning and residue removal plots. ZTW sown with surface retention of rice crop residue increased the organic carbon by 0.36–0.42% and the soil moisture content by 13.4–23.6% over CTW without residue load. Similarly, ZTW sown with Happy seeder with full residue enhanced alkaline phosphatase activity from 95.3 µg TPF g^−1^ soil 24 h^−1^ in 2018–2019 to 98.6 µg TPF g^−1^ soil 24 h^−1^ in 2019–2020 over control plots. Likely, microbial population and enzymatic activity showed strong positive correlation under variable residue retention practices. However, increased microbial population reduced the soil pH from 7.49 to 7.27 under ZTW with residue retention plots. The wheat yield enhanced by 9.8–11.3% during 2018–2019 and 2019–2020 under ZTW with Happy seeder with full residue load over residue burning and residue removal plots. ZTW sown with Happy seeder under full residue retention, achieved maximum net return 43.16–57.08 × 10^3^ ₹ ha^−1^) and B-C ratio (1.52 to 1.70) over CTW without residue. Therefore, rice residue needs to be managed by planting wheat using appropriate machinery under ZT for sustaining higher productivity in RWCS and improve soil health and environment under IGP regions.

## Introduction

The primary cropping system in the north-western (N-W) Indo-Gangetic Plains (IGP) of India is the rice–wheat cropping system (RWCS), which covers 4.1 million hectares, primarily in the states of Punjab, Haryana, Uttarakhand, and western Uttar Pradesh and produces 34 million tonnes of rice crop residue^1^. According to recent estimates, Southeast Asian countries produce 150 MT of rice residues each year^2,3^. Harvesting and threshing of coarse rice are largely and commonly done by combine harvesters ending into leftover residues behind (in narrow strips or gluts), particularly when these machines are not attached with spreader. The window for disposal or use of rice residues is very constrained between rice harvest and the sowing of rabi (october to november) crops like wheat, potatoes, or vegetables. As a result, 80% of the total rice residue produced annually is burnt fully or partially by the farmers^4,5^. And burning of rice and wheat residue contributes about 42% of total green house gas emmision (GHGs) of the country^6^. Not only GHGs, aromatic hydrocarbons, volatile organic compounds, and fine inhalable particles are released during residue burning^7^, which are the factors contributing to the formation of atmospheric brown cloud (ABC), having an impact on the air quality index and atmospheric visibility in several Asian countries^8^. However, wheat crop residues are not burnt at scale as these are being used for feeding the livestock. Despite the importance of RWCS, over the past three decades, signs of yield stagnation, groundwater depletion, and declining soil health have made the sustainability of the system as the most pressing concern^9^. The most obvious concern for the threatening sustainability of RWCS in the IGPs in the context of multicontemporary challenges is the environmental pollution caused by residue burning^3^. The N-W Indian states burn 23 million tonnes of rice residue each year, and collecting and storing this residue is neither practical nor cost-effective^10,11^. This results not only into a serious environmental pollution but also into huge loss of nutrients^12–15^. Burning of rice residues reduces the amount of C added as residue into the soil's organic C pool because it leaves high C footprints^3^. Crop residues are rich source of plant nutrients which are released in soil on decomposition by beneficial microbes. As a result, returning crop residue to the soil rather than burning it helps to improve several soil quality parameters. In order to sustain the health of the soil in the RWCS in NW India, it is necessary to manage rice residue in a way that is affordable, environmentallybeing, and logistically possible.

In general, crop residues contain about 40 to 45% of carbon, which if returned back to soil, the soil microbes utilized it to increase soil organic matter and thus returning of crop residues reduces organic carbon loss^16^. Soil organic carbon directly influences biological properties of soil^17,18^. Microorganisms play a significant role in nutrient availability and the development of a number of soil health indices, knowledge of soil biological characteristics is crucial for sustainability^15,19–21^. Total microbial count, diazotrophs, actinomycetes and enzymatic activities like dehydrogenase and alkaline phosphatase are some of the major soil quality indicators^22^. By converting organic matter's organic form to an easily-accessible inorganic form, soil bacteria aid in the breakdown of organic matter^23^. Recycling of crop waste is essential to return organic matter into the soil^24–27^. Actinomycetes help in cellulose and hemicellulose compound degradation in rice residue. Diazotrophs are nitrogen fixers; which utilize the carbon from residue and release nitrogen for the growing crop. As per Costa et al.^28^ and Shahrayini et al.^29^, reduced tillage and stubble retention result in abundance of diazotrophs as decreased levels of soil disturbance promote good soil pore network which helps interaction of stubble decomposing organisms and nitrogen fixers. In order to develop and maintain lower O_2_ tension, which is necessary for many N fixers using diazotrophs, this increases the number of soil microsites with organic carbon and improves soil macro-agregates^30^. A sensitive indicator of soil quality, dehydrogenase enzyme is an oxide-reductase enzyme that is found in all living microbial cells and is essential for sustaining soil health and fertility^31^. According to Rana et al.^32^ alkaline phosphatase activity is primarily of microbial origin and can be utilised as a short-term indication of changes in microbial activity. The breakdown process and biochemical processes are accelerated by soil enzymes, which also release plant nutrients^33–35^.

The most effective intervention needed to improve the C sustainability and resilience of RWCS is to switch from burning rice residue in-situ to retaining it and/or incorporating it into the field. Varied levels of residue retention on the surface or incorporation, coupled with residue removal and partial or full residue burning, are the resultant of different tillage and residue management strategies^12,36–38^. This ultimately has an impact on the processes and activities of soil microorganisms^39,40^. Crop residue burning raises soil temperature, which in turn leads to a decline in microbial populations. When paddy straw is burnt in the field a major change undergoes in soil microbial population. However, limited information is available on soil microbial dynamics under residue burning in RWCS of north-west IGP of Haryana situations. For in-situ residue management, a recent technological advancement with the development of second generation machinery such as the Happy Seeder is critical, which is a modest and viable rice residue management (RRM) option, capable of directly drilling wheat seed in rice stubbles without tillage. Happy Seeder has enabled direct wheat seeding while cutting heavy loads of loose and anchored rice residue into mulch^41^. In comparison to CT, residue retention as mulch in wheat establishment using Happy Seeder technology reduces C and energy footprints by 14.1 and 12.9%, respectively^42^. Therefore, a focused research efforts on these issues are required to generate viable and sustainable options for residue management and improving soil fertility and productivity of the strained rice–wheat system. Thus, the current study was carried out to assess the effect of different Happy seeder based residue management options like residue retention, incorporation, and burning on changes in microbial dynamics, soil organic carbon, enzymatic activities,and productivity of wheat under rice–wheat system.

## Material and methods

### Study area

The experiment was carried out in sandy loam soils at CCS Haryana Agricultural University's Regional Research Station in Karnal, India [290 43′ 41″ North and 760 58′ 50″ East]. The study soils had 57.5%, 23.4%, and 18.2% sand, silt and clay, respectively. Prior to commencing the study soil was sampled from the entire experimental field at 0 to 15 cm depth, and was analysed subsequently after making a composite sample. The soil's initial pH value was 7.74 (1:2.5 soil–water ratio) with elctrical conductivity (EC) of 0.22 dS m^−1^, 1.52 g cm^−3^ bulk density, 0.34% soil organic carbon, 134.2 kg ha^−1^ KMnO_4_ oxidizable N, 13.74 kg ha^−1^ NaHCO_3_ extractable phosphorus (P) and 280.4 kg ha^−1^ 1.0 N NH_4_OAc exchangeable potassium (K).

### Experimental details and field management

**Figure 1:**
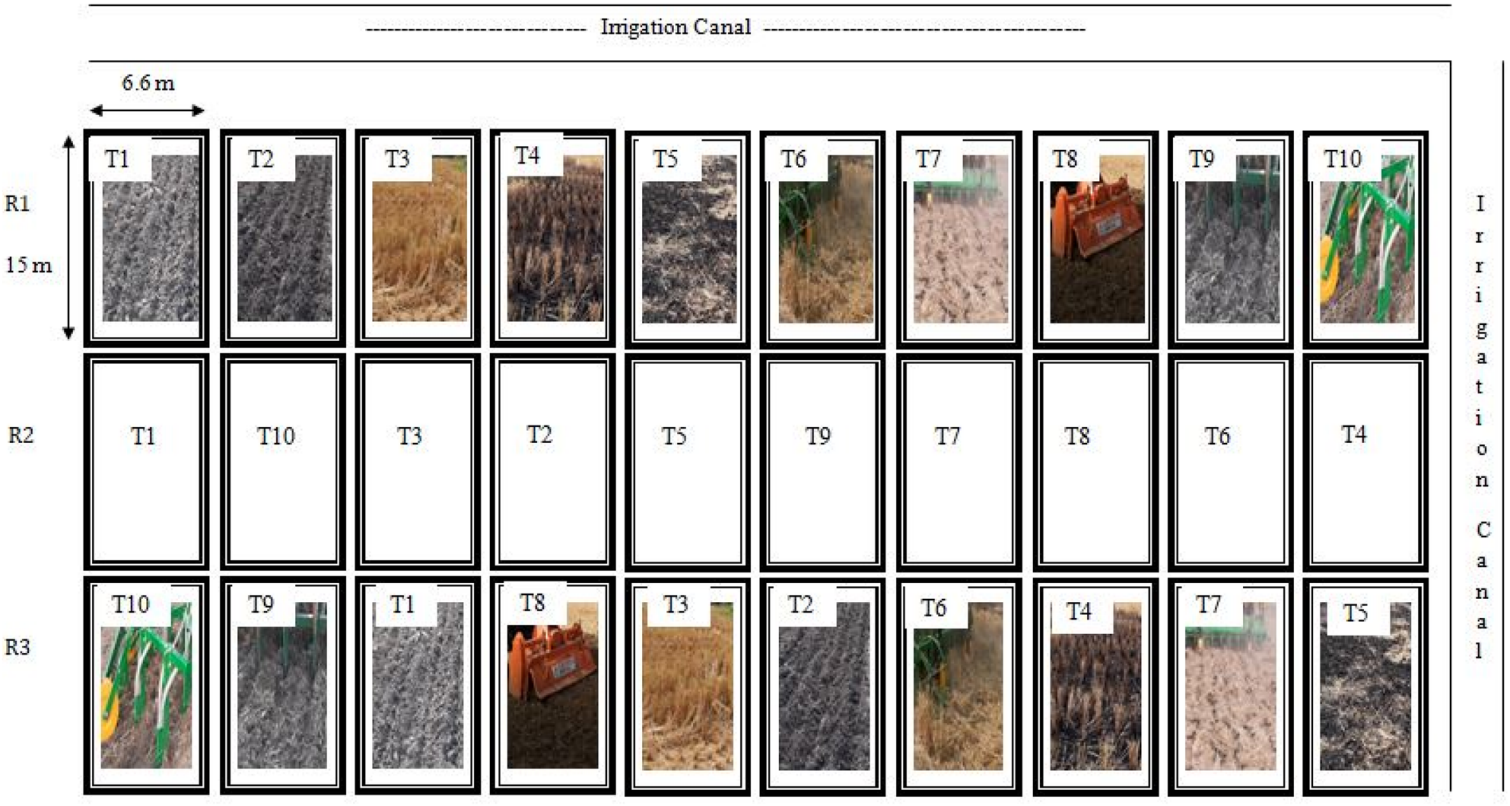
Layout of the experimental field.

The current study consisted of 10 treatments viz., conventional tillage wheat (CTW), CTW drill sown (without burning), zero tillage wheat (ZTW) with anchored stubbles, ZTW after partial burning, ZTW after full residue burning, ZTW with Happy seeder (HS) in full residue load, ZTW with Happy seeder after using chopper and spreader (full residue load), CTW broadcast sown with rotavator, CTW drill sown after using chopper, spreader & rotavator, and CTW spatial drill sown, were laid out using randomized complete block design (RCBD) with three replications (Fig. 1). Happy seeder is a tractor-mounted machine that cuts and lifts rice straw, sows wheat into the soil, and deposits the straw over the sown area as mulch (https://www.cimmyt.org/news/happy-seeder-can-reduce-air-pollution-and-greenhouse-gas-emissions-while-making-profits-for-farmers/). It consists of a straw management rotor for cutting the previous crop residues and a zero till drill for sowing of next crop. Flail type straight blades are mounted on the straw management rotor which cuts (hits/shears) the standing stubbles/loose straw coming in front of the sowing tyne and clean each tyne twice in one rotation of rotor for proper sowing. The flails push the residues as surface much between the seeded rows.

The net plot size of the individual plots was 15 m × 6.6 m = 99 m^2^. Brief details of treatments with procedure of residue incorporation and burning are given in Table 1. Burning of crop residues was done by putting the loose straw on fire on the field itself after harvesting of the rice crop and before applying the pre-sowing irrigation in the field. For the treatment of residue removal, loose straw was collected from the field manually and taken out. Before planting the next wheat crop, a chopper-cum-spreader machine was used to uniformly spread and chop up loose straw across the field. Pre-sowing irrigation was given before sowing of wheat crop. Different levels of residue burning (no burning, partial burning, or complete burning) were maintained in zero-tillage plots with anchored stubbles according to treatments, and seeding was done by drilling with a zero-till drill (T3, T4, T5). In zero tillage plots with full residue retention (chopped or unchopped), sowing was done using the Happy seeder machine which directly sows the seed in loose stubbles (T6, T7). In conventional tillage plots without crop residues (after residue burning or removal), two harrowing and one-time rotavator followed by planking were used for field preparation (T1, T2). Sowing was done by using the seed-cum-fertilizer drill. In conventional tillage plots with full residue retention (chopped), the residues were mixed in soil by using the rotavator twice. Sowing was done by broadcast of seed and fertilizers and mixing with rotavator (T8), using normal zero-tillage drill (T9) and spatial drill (T10) to avoid heap collection of chopped crop residues in rows, as per different treatments. All the sowing machines were having seed-cum-fertilizer drill application mechanism, hence basal dose of fertilizers was also applied through these machines in all the treatments. Cutting, chopping, spreading and tillage operations were done 1–2 days before sowing.

**Table 1:** Details of different treatments imposed.

S. no	Treatment	Details
T1	Conventional tillage wheat (CTW) drill sown after full residue burning	Anchored stubbles (the stubbles remaining intact in the field after mechanical harvest of rice crop) were cut with shrub master machine. Burning of crop residues was done by putting the loose straw on flame fires in the field itself after harvesting of the rice crop and before applying the pre-sowing irrigation in the field. For field preparation, two harrowing and one-time rotavator followed by planking were used. Sowing was done by using the zero-tillage drill
T2	CTW drill sown after removal of residues (without burning)	Anchored stubbles were cut with shrub master machine. Loose straw was collected from the field manually and taken out. Two harrowing and one-time rotavator followed by planking were used for field preparation. Sowing was done by using the zero-tillage drill
T3	Zero tillage wheat (ZTW) with anchored stubbles	Loose straw was removed manually and only anchored stubbles (30% residue) were maintained. Sowing was done by directly drilling with Zero-till drill under no-till situations in anchored stubbles
T4	ZTW after anchored stubbles partial burning	Loose straw was removed manually, and partial burning of anchored stubbles was done by flame fires. In no-till situation, sowing was carried out by directly drilling with a zero-till drill
T5	ZTW after full residue burning	Complete burning of all crop residues including anchored stubbles cut with shrub master was done. Flame fires were used for burning the residues. In no-till situation, sowing was carried out by directly drilling with a zero-till drill
T6	ZTW with Happy seeder (HS) in full residue load	Full residues (6 t ha^−1^) was maintained on surface under no-till situations. There were anchored stubbles and loose straw uniformly spread in the whole plot. The Happy seeder machine was used for sowing the seed in loose stubbles
T7	ZTW with Happy seeder after using chopper & spreader (full residue load)	Full residues (6 t ha-1) was maintained on surface under no-till situations. These residues were cut into pieces and spread uniformly by use of Chopper and Spreader machine before sowing of the succeeding wheat crop. The Happy seeder machine was used for sowing the seed in loose stubbles
T8	CTW broadcast sown with rotavator after using chopper & spreader (full residue load)	Full residue of 6 t ha^−1^ was maintained. Residues were cut into pieces and spread uniformly by use of Chopper and Spreader machine. Residues were mixed in soil by using the rotavator twice followed by planking. Sowing was done by broadcast of seed along with fertilizer and mixing with second pass of rotavator
T9	CTW drill sown after using chopper, spreader & rotavator (full residue load)	Full residue of 6 t ha^−1^ was maintained. Residues were cut into pieces and spread uniformly by use of Chopper and Spreader machine. Residues were mixed in soil by using the rotavator twice followed by planking. Sowing was done by using zero-till drill
T10	CTW spatial drill sown after using chopper & spreader (full residue load)	Full residue of 6 t ha^−1^ was retained in the field. Residues were cut into pieces and spread uniformly by use of Chopper and Spreader machine. The residues were mixed in soil by using the rotavator twice followed by planking. Sowing was done by using Spatial drill, which avoids collection of residue in heaps over the sowing rows

Rice variety HKR-47 transplanted in the field on 1st July was harvested on 23rd October and 25th October during kharif seasons of 2018 and 2019, respectively. Cultivation practices were followed as per package and practices of Haryana Agricultural University, Hisar (India). Sowing of wheat was carried out in zero-till plots using a Happy seeder and a ZT seed-cum-fertilizer drill equipped with inverted T-tynes. CT plots with residues were sown with the same ZT seed-cum-fertilizer drill, and residues were manually broadcasted and mixed with a rotavator before sowing. Wheat variety HD-2967 was sown on 4th November and 17th November during 2018 and 2019, respectively, using a seed rate of 100 kg ha^−1^. Recommended quantities of phosphorus (60 kg P_2_O_5_ ha^−1^) and nitrogen (150 kg N ha^−1^) were applied during both growing seasons. Phosphorus (100%) and 50% of N were given as the basal dose. After first irrigation, remaining 50% of nitrogen was top dressed as urea in two splits. Urea and DAP were the sources of the nitrogen and phosphorus, respectively. Based on soil indications (gravimetric method) and visual plnat symptoms, total of 7 irrigations in first year and 5 irrigations in second year were given in a season from sowing to the harvest of the crop including one pre-sowing irrigation. Herbicide, mesosulfuron + iodosulfuron 12 + 2.4 g ha^−1^ was used to control weeds at 35 days after sowing (DAS) as a spray in a water volume of 500 L ha^−1^ using a knapsack sprayer equipped with a flat fan nozzle. Other management techniques were employed as per recommendations of the State University.

### Data curation and analysis

The soil samples were taken from the field before and immediately after the treatments were assigned. At harvest of the termination of the experiment (after two years), soil samples were again taken from each plot (two samples from each plot were combined to make one composite sample) at a depth of 0 to 15 cm. A post-hole auger was used for drawing composite samples and determined soil moisture content gravimetrically (w/w) at 75 days after sowing and at maturity.

Likewise, with a post-hole auger, soil samples were taken from each plot at two different stages of wheat growth, i.e., 75 DAS and at crop maturity, for the estimation of the microbial community and enzyme activity. Fresh soil samples were then crushed, sieved through a 2 mm sieve, and allowed to air dry before being utilised for chemical analysis. The methodology used for soil analysis on chemical and biological parameters are provided in Table 2.

**Table 2:** Methods used for different soil biochemical analysis.

Soil biochemical properties	Method used	References
pH	pH meter	Datta et al. (1997)
EC	electronic EC meter	Datta et al. (1997)
OC	Wet oxidation method	Walkley and Black (1934)
Available N	Kjeldahl method	Subbiah and Asija (1956)
Available P	Olsens method	Olsen et al. (1954)
Available K	Flame photometer method	Hanway and Heidel (1952)
Soil microbial count (10^7^ cfu/g soil)	Standard serial dilution technique on soil extract media	Wright (1933)
Diazotrophic count (10^4^ cfu/g soil)	Standard serial dilution technique on nutrient agar media	Wright (1933)
Actinomycetes count (10^5^ cfu/g soil)	Standard serial dilution technique on Kenknights media	HiMedia (2009)
Dehydrogenase activity	Triphenylformazon through reduction of 2,3,5 triphenyltetrazolium chloride	Casida et al. (1964)
Alkaline phosphatase activity	n-nitrophenyl method	Tabatabai and Bremner (1969)

To determine the impact of various crop establishment and residue management techniques on the chemical and biological activities of the soil, analysis of variance (ANOVA) was carried out for statistical evaluation of different treatments. To determine the significant differences between various treatments, Duncan's multiple range test (DMRT) was used in conjunction with the standard error of mean (SEm ±) and least significant difference (LSD) computations^43^.

## Results

### Influence of rice residue management alternatives soil chemical properties

Rice residue management techniques in wheat influended the soil organic carbon during 2019–2020 (Table 3). Both initial and final values of soil pH showed non-significant differences among the various treatments. Surface retention of rice residue resulted in higher organic carbon content than residue incorporation or CTW without residue (burning/removal). ZTW sown with surface retention of rice crop residue increased the organic carbon (0.36–0.42%) than CTW without residue (0.32–0.33%) and CTW with full residue incorporation (0.39–0.40%). In comparison to conventional tillage (T2), full crop residue retention on the soil surface with ZTW planted with HS (T6) resulted in an increase in organic carbon in the upper 0–15 cm soil layer by 23.8%.

**Table 3:** Soil pH and soil organic carbon under rice residue management and crop establishment methods under rice–wheat cropping system (initial and after 2 years cycle).

Treatment		Soil pH		Soil OC (%)	
		Initial	Final	Initial	Final
T1	CTW drill sown after full residue burning	7.73 ± 0.07a	7.74 ± 0.09a	0.34 ± 0.01a	0.33 ± 0.02d
T2	CTW drill sown after removal of residues (without burning)	7.74 ± 0.02a	7.75 ± 0.16a	0.34 ± 0.02a	0.32 ± 0.02d
T3	ZTW with anchored stubbles	7.76 ± 0.11a	7.43 ± 0.14a	0.35 ± 0.02a	0.38 ± 0.01abc
T4	ZTW after anchored stubbles partial burning	7.77 ± 0.11a	7.44 ± 0.11a	0.34 ± 0.02a	0.37 ± 0.02bc
T5	ZTW after full residue burning	7.79 ± 0.17a	7.49 ± 0.09a	0.34 ± 0.01a	0.36 ± 0.02cd
T6	ZTW with Happy seeder in full residue load	7.78 ± 0.04a	7.30 ± 0.22a	0.35 ± 0.02a	0.42 ± 0.01a
T7	ZTW with Happy seeder after using chopper & spreader (full residue load)	7.75 ± 0.06a	7.27 ± 0.30a	0.34 ± 0.02a	0.41 ± 0.01a
T8	CTW broadcast sown with rotavator after using chopper & spreader (full residue load)	7.76 ± 0.07a	7.50 ± 0.08a	0.35 ± 0.03a	0.39 ± 0.02abc
T9	CTW drill sown after using chopper, spreader & rotavator (full residue load)	7.78 ± 0.05a	7.51 ± 0.23a	0.34 ± 0.01a	0.40 ± 0.01abc
T10	CTW with spatial drill after using chopper & spreader (full residue load)	7.79 ± 0.06a	7.50 ± 0.07a	0.34 ± 0.02a	0.40 ± 0.01ab

The values within columns with different letters differed significantly with each other. For all variables n = 3 ± standard error of mean.

### Influence of rice residue management alternatives on soil moisture content

In both of the rabi cropping seasons (2018–2019 and 2019–2020), the rice residue management techniques had a substantial impact on soil moisture content (SMC) in wheat (Table 4). All the zero tillage (ZT) treatments retained higher SMC during the entire crop growth period. The SMC was higher at maturity than 75 DAS during both seasons. ZTW sown with Happy seeder (HS) after using chopper and spreader got maximum SMC (17.93 and 20.37% in 2018–2019 and 2019–2020, respectively) which was statistically similar with ZTW sown with HS with full residue load and significantly higher than CTW without residue (burning/removal) and CTW with full residue incorporation. Thus, ZTW sown with Happy seeder (HS) after using chopper and spreader (T7) enhanced soil moisture content by 16.94–23.60% and 13.44–16.20% at 75 DAS and at harvest, respectively over CTW residue removal plots.

**Table 4:** Effect of rice residue management and wheat crop establishment methods on soil moisture content (%) in wheat crop under rice–wheat cropping system (2018–2019 and 2019–20).

Treatment		Soil moisture content (%) at 0-10 cm soil depth			
		75 DAS		Harvest	
		2018–2019	2019–2020	2018–2019	2019–2020
T1	CTW drill sown after full residue burning	11.82 ± 0.81b	11.64 ± 0.59b	15.63 ± 0.68b	17.20 ± 1.47b
T2	CTW drill sown after removal of residues (without burning)	11.62 ± 0.31b	11.57 ± 0.08b	15.52 ± 1.04b	17.07 ± 1.51b
T3	ZTW with anchored stubbles	12.64 ± 0.49b	12.62 ± 0.64ab	17.00 ± 1.00ab	19.73 ± 0.83a
T4	ZTW after anchored stubbles partial burning	12.58 ± 0.60b	12.57 ± 0.30ab	16.94 ± 1.10ab	19.72 ± 0.67a
T5	ZTW after full residue burning	12.21 ± 0.57b	12.29 ± 0.36b	16.50 ± 0.50ab	19.00 ± 1.15ab
T6	ZTW with Happy seeder in full residue load	15.18 ± 0.38a	13.86 ± 0.53a	17.89 ± 0.75a	20.32 ± 1.44a
T7	ZTW with Happy seeder after using chopper & spreader (full residue load)	15.21 ± 0.69a	13.93 ± 0.17a	17.93 ± 1.07a	20.37 ± 1.69a
T8	CTW broadcast sown with rotavator after using chopper & spreader (full residue load)	11.98 ± 1.08b	12.00 ± 0.51b	16.23 ± 0.96ab	18.50 ± 1.32ab
T9	CTW drill sown after using chopper, spreader & rotavator (full residue load)	12.11 ± 1.51b	12.18 ± 0.64b	16.28 ± 0.89ab	18.63 ± 2.02ab
T10	CTW with spatial drill after using chopper & spreader (full residue load)	12.15 ± 0.60b	12.25 ± 0.35b	16.38 ± 0.69ab	18.74 ± 1.75ab

The values within columns with different letters differed significantly with each other. For all variables n = 3 ± standard error of mean.

### Influence of rice residue management alternatives on microbial counts

In both of the cropping seasons (2018–2019 and 2019–2020), the effect of rice residue management had a substantial impact on the soil microbial characteristics in wheat (Table 5). Zero tillage treatments performed well in case of soil biological activity. All the microbial populations were more at 75 DAS as compared to at harvest during both seasons. ZTW sown with Happy seeder (HS) after using chopper and spreader got maximum soil microbial count at 75 DAS (94.9 × 10^7^ and 99.8 × 10^7^ cfu/g soil in 2018–2019 and 2019–2020, respectively) followed by ZTW sown with HS with full residue load, which were higher than CTW without residue (burning/removal) and CTW with full residue incorporation. Thus, ZTW sown with Happy seeder after using chopper and spreader enhanced soil microbial count by 23.7–38.3% and 47.9–60.4% at 75 DAS and at harvest, respectively, over residue burning and residue removal plots.

**Table 5:** Effect of crop establishment methods and rice residue management on soil microbial count and diazotrophic count under rice–wheat cropping system (2018–2019 and 2019–20).

Treatment	Soil microbial count (10^7^ cfu/g soil)				Diazotrophic count (10^4^ cfu/g soil)			
	75 DAS		Harvest		75 DAS		Harvest	
	2018–2019	2019–20	2018–2019	2019–20	2018–2019	2019–20	2018–2019	2019–20
T1	71.7 ± 0.87e	82.8 ± 1.44e	58.7 ± 2.25d	70.1 ± 1.73d	49.2 ± 2.31ef	61.4 ± 3.18d	41.9 ± 1.53ef	47.7 ± 2.25d
T2	68.6 ± 2.02f	80.7 ± 2.60e	55.6 ± 2.78d	60.6 ± 1.73e	47.8 ± 2.18f	58.0 ± 1.44e	36.6 ± 1.15f	42.0 ± 1.44e
T3	88.4 ± 2.60b	96.6 ± 3.01abc	74.5 ± 2.73b	80.6 ± 3.06b	58.0 ± 1.15b	75.3 ± 1.44b	50.8 ± 3.51bc	65.1 ± 2.25b
T4	82.8 ± 1.44 c	94.7 ± 1.45bcd	69.8 ± 1.44c	77.4 ± 1.73bc	54.7 ± 1.33c	66.9 ± 1.44c	49.9 ± 1.15cd	56.4 ± 5.41c
T5	81.2 ± 1.44c	90.8 ± 1.44d	68.2 ± 1.44c	74.4 ± 1.73cd	53.8 ± 2.89cd	66.4 ± 1.44c	45.4 ± 2.31cde	52.6 ± 2.42cd
T6	94.1 ± 2.60a	98.6 ± 4.37ab	88.0 ± 1.73a	89.3 ± 3.14a	61.1 ± 2.31ab	78.2 ± 1.44ab	56.0 ± 2.30ab	71.0 ± 1.53a
T7	94.9 ± 1.44a	99.8 ± 1.44a	89.2 ± 2.12a	89.6 ± 1.90a	62.1 ± 1.97a	79.3 ± 1.44a	58.2 ± 5.28a	72.7 ± 0.15a
T8	80.9 ± 1.44c	92.8 ± 1.44cd	67.8 ± 2.77c	73.3 ± 1.73cd	51.8 ± 1.15cde	65.3 ± 3.18c	44.8 ± 2.00de	52.8 ± 1.44cd
T9	80.3 ± 1.49cd	91.4 ± 1.44d	67.3 ± 1.49c	72.5 ± 1.73d	50.4 ± 1.15def	64.8 ± 1.44c	44.5 ± 1.15de	51.8 ± 1.44cd
T10	77.7 ± 1.44d	92.1 ± 1.44cd	65.8 ± 4.74c	72.2 ± 1.73d	50.4 ± 2.31def	64.1 ± 3.28cd	42.7 ± 2.00e	51.4 ± 1.44cd

The values within columns with different letters differed significantly with each other. For all variables n = 3 ± standard error of mean.

T1, CTW drill sown after full residue burning; T2, CTW drill sown after removal of residues (without burning); T3, ZTW with anchored stubbles; T4, ZTW after anchored stubbles partial burning; T5, ZTW after full residue burning; T6, ZTW with Happy seeder in full residue load; T7, ZTW with Happy seeder after using chopper & spreader (full residue load); T8, CTW broadcast sown with rotavator after using chopper & spreader (full residue load); T9, CTW drill sown after using chopper, spreader & rotavator (full residue load); T10, CTW with spatial drill after using chopper & spreader (full residue load).

Similarly, diazotrophic count at 75 DAS was higher in ZTW sown with HS after using chopper and spreader (62.1 × 10^4^ and 79.3 × 10^4^ cfu/g soil in 2018–2019 and 2019–2020, respectively). It was statistically similar with ZTW sown with HS in uniformly spread full residue load and higher as compared to CTW with full residue incorporation and CTW without residue (Table 5). The similar trend was followed at harvest. Burning of crop residue further reduced the diazotrophic count and attained lowest numbers. Therefore, ZTW sown with Happy seeder after using chopper and spreader enhanced diazotrophic count by 29.9–36.7% and 59.0–73.1% at 75 DAS and at harvest, respectively, over residue burning and residue removal plots.

Actinomycetes count at 75 DAS was also higher in ZTW sown with happy seeder after using chopper and spreader (68.9 × 10^5^ and 64.1 × 10^5^ cfu/g soil in 2018–2019 and 2019–2020, respectively). It was statistically at par with ZTW sown with Happy Seeder in full residue load (Table 6). CTW without residue (burning/removal) got lowest actinomycetes count in ZTW and CTW with or without residue. However, ZTW sown with Happy seeder after using chopper and spreader enhanced diazotrophic count by 37.0–39.2% and 47.3–54.2% at 75 DAS and at harvest, respectively, over residue burning and residue removal plots.

**Table 6:** Effect of rice residue management and wheat crop establishment methods on actinomycetes count of wheat under rice–wheat cropping system (2018–2019 and 2019–20).

S. N	Treatment	Actinomycetes count (10^5^ cfu/g soil)			
		75 DAS		At harvest	
		2018–2019	2019–2020	2018–2019	2019–2020
T1	CTW drill sown after full residue burning	49.5 ± 1.36e	51.8 ± 1.44d	32.8 ± 1.42d	34.3 ± 0.88c
T2	CTW drill sown after removal of residues (without burning)	49.7 ± 3.73f	46.8 ± 1.44e	33.8 ± 2.04cd	36.7 ± 1.45c
T3	ZTW with anchored stubbles	57.8 ± 2.90b	58.0 ± 3.64b	39.8 ± 1.41b	45.9 ± 1.53b
T4	ZTW after anchored stubbles partial burning	56.1 ± 2.18e	55.4 ± 1.44bc	38.2 ± 1.16cd	44.4 ± 1.15b
T5	ZTW after full residue burning	55.2 ± 1.76f	51.0 ± 1.44d	34.0 ± 1.11cd	43.5 ± 2.89b
T6	ZTW with Happy seeder in full residue load	67.6 ± 1.75a	63.8 ± 2.33a	47.4 ± 1.68a	52.0 ± 1.73a
T7	ZTW with Happy seeder after using chopper & spreader (full residue load)	68.9 ± 0.88a	64.1 ± 2.34a	48.3 ± 2.27a	52.9 ± 1.74a
T8	CTW broadcast sown with rotavator after using chopper & spreader (full residue load)	53.3 ± 1.76c	53.9 ± 2.61cd	34.5 ± 1.02cd	43.0 ± 1.15b
T9	CTW drill sown after using chopper, spreader & rotavator (full residue load)	52.7 ± 1.45cd	57.1 ± 2.80bc	32.8 ± 2.16d	42.4 ± 1.15b
T10	CTW with spatial drill after using chopper & spreader (full residue load)	52.5 ± 2.99d	56.0 ± 2.67bc	33.0 ± 2.31d	42.2 ± 1.15b

The values within columns with different letters differed significantly with each other. For all variables n = 3 ± standard error of mean.

### Influence of rice residue management alternatives on wheat productivity

**Figure 2:**
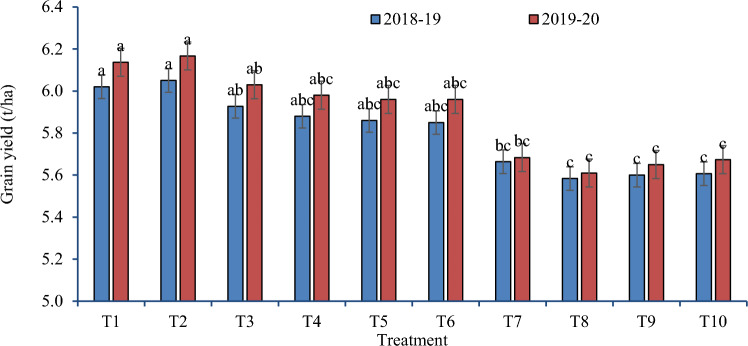
Effect of rice residue management and wheat crop establishment methods on grain yield of wheat under rice–wheat cropping system (2018–2019 and 2019–2020).

**Figure 3:**
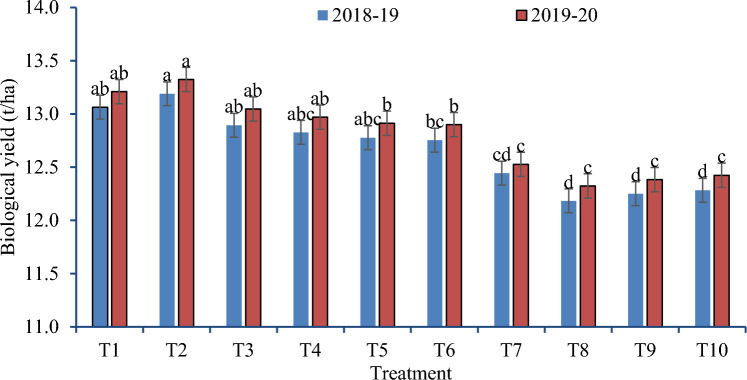
Effect of rice residue management and wheat crop establishment methods on biomass yield of wheat under rice–wheat cropping system (2018–2019 and 2019–2020).

The rice residue management options differed significantly with wheat grain and biomass yields during both cropping seasons (Figs. 2, 3). ZTW without full residue retention (after full residue burning, in anchored stubbles without or with partial burning) and ZTW with happy seeder in uniformly spread full residues produced grain (Fig. 2) and biomass yields (Fig. 3) similar to traditional establishment methods of CTW without residue retention (burning or removal) and higher than CTW with full residue incorporation after chopper and spreader (sown with seed drill, spatial drill or broadcast). However, ZTW sown with Happy seeder with or without using chopper and spreader enhanced wheat grain yield and biomass yields by 9.8–11.3% and 7.4–9.6% during 2018–2019 and 2019–2020, respectively, over residue burning and residue removal plots. ZTW with Happy seeder after chopper & spreader produced lower grain and biomass yield than other methods of ZTW with few exceptions. Performance of Happy seeder sown ZTW after chopper and spreader was similar to Happy seeder sowing in evenly spread residues during 2018–2019. All the methods of crop establishment under ZT/CT after use of chopper and spreader produced lower grain and biomass yields than CTW after residue removal/ burning and ZTW with or without residue retention.

### Influence of rice residue management alternatives on economics of the various treatments

**Figure 4:**
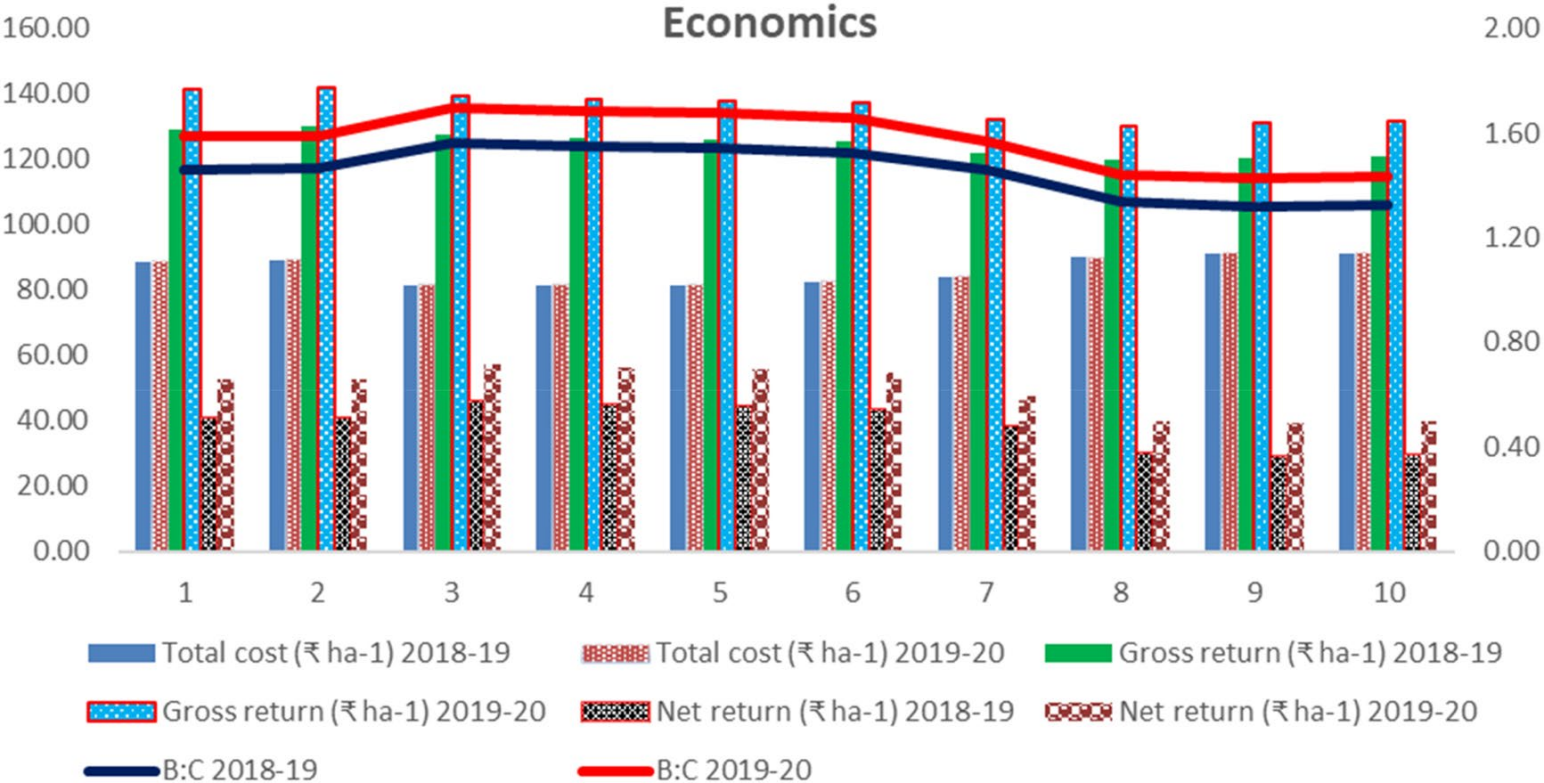
Effect of rice residue management and wheat crop establishment methods on economics.

The cost of cultivation, gross and net returns and benefit–cost ratio (B:C) of wheat worked out under rice residue management in no-till wheat under rice–wheat cropping system during two cropping seasons of 2018–2019 and 2019–2020 have been given in the Fig. 4. ZTW without full residue retention (after full residue burning, in anchored stubbles without or with partial burning) incurred the lowest cost of cultivation (81.49–81.93 × 10^3^ ₹ ha^−1^) followed by ZTW sown with Happy seed under full residue retention (Fig. 4) which were lower than CTW without residue (removal or burning) and crop establishment methods after using chopper and spreader (drill sown, broadcast, spatial drill) (Fig. 4) during both the crop seasons. Similarly, ZTW with partial residue (after full residue burning, in anchored stubbles without or with partial burning) and ZTW sown with Happy seeder under full residue retention, achieved maximum net return (43.16–57.08 × 10^3^ ₹ ha^−1^) and B-C ratio (1.52 to 1.70) over CTW without residue and crop establishment methods after using chopper and spreader (drill sown, broadcast, spatial drill) during both the crop seasons of 2018–2019 and 2019–2020, respectively.

### Influence of rice residue management alternatives on enzymatic activity

**Figure 5:**
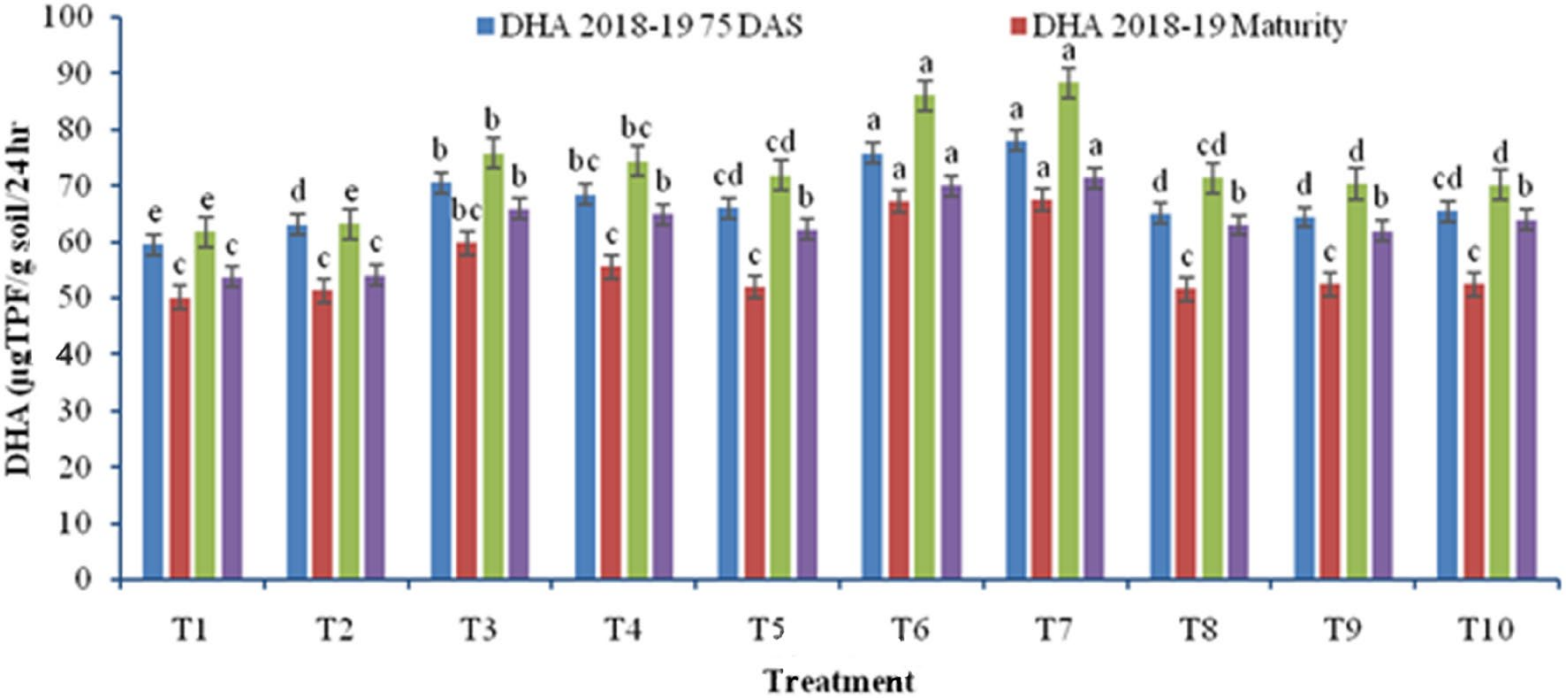
Effect of rice residue management and wheat crop establishment methods on dehydrogenase activity (DHA) of wheat under rice–wheat cropping system (2018–2019 and 2019–2020). The values within same colour columns with different letters differed significantly with each other.

In both of the cropping seasons, the effect of rice residue management had a substantial impact on the enzymatic activity in wheat grown in a rice–wheat system (Fig. 5). Compared to residue retention, residue burning reduced the dehydrogenase activity at both stages of wheat crop. ZTW sown with HS using chopper and spreader in full residue load released highest dehydrogenase activity at 75 DAS (78.0 µgTPF g^−1^ soil 24 h^−1^ in 2018–2019; 88.4 µg TPF g^−1^ soil 24 h^−1^ in 2019–2020), which was 19.1–23.4% more than CTW drill sown after full residue removal during the study seasons. The treatments T6 and T7 having ZTW sown with HS were statistically similar when wheat was sown after uniformly spread full residues or after chopper & spreader. A similar trend was observed at harvest. ZTW with full or partial residue burning reduced the enzymatic activity as compared to ZTW with full residue retention. CTW with full residue burning further reduced the enzyme activity as compared to CTW without residue.

**Figure 6:**
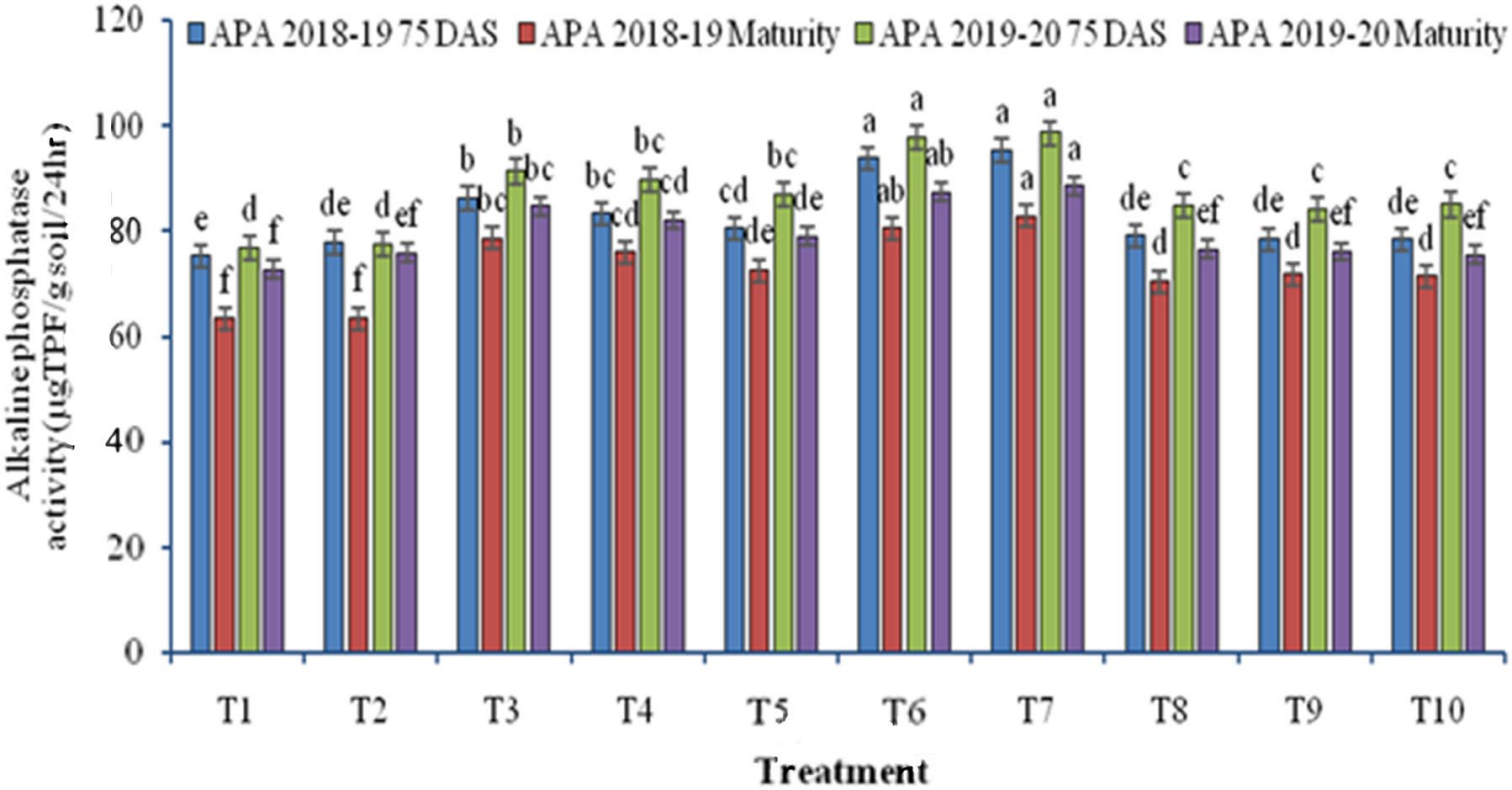
Effect of rice residue management and wheat crop establishment methods on alkaline phosphatase activity (APA) of wheat under rice–wheat cropping system (2018–2019 and 2019–2020).

Alkaline phosphatase activity significantly differed with different rice residue management methods during 2018–2019 and 2019–2020 cropping years (Fig. 6). Residue retention on soil surface got higher alkaline phosphatase activity than residue incorporation or removal. ZTW sown with Happy seeder after using chopper and spreader with full residue load got maximum alkaline phosphatase activity at 75 DAS (95.3 µg TPF g^−1^ soil 24 h^−1^ in 2018–2019; 98.6 µg TPF g^−1^ soil 24 h^−1^ in 2019–2020). It was at par with ZTW sown with HS with full residue load and higher than CTW drill sown with full residue removal. Conventionally tilled plots with full residue burning further reduced the alkaline phosphatase activity at 75 DAS (26.6–28.6%) as compared to ZTW sown with HS after using chopper and spreader with full residue load.

### Relationship between microbial populations and enzymatic activity

**Figure 7:**
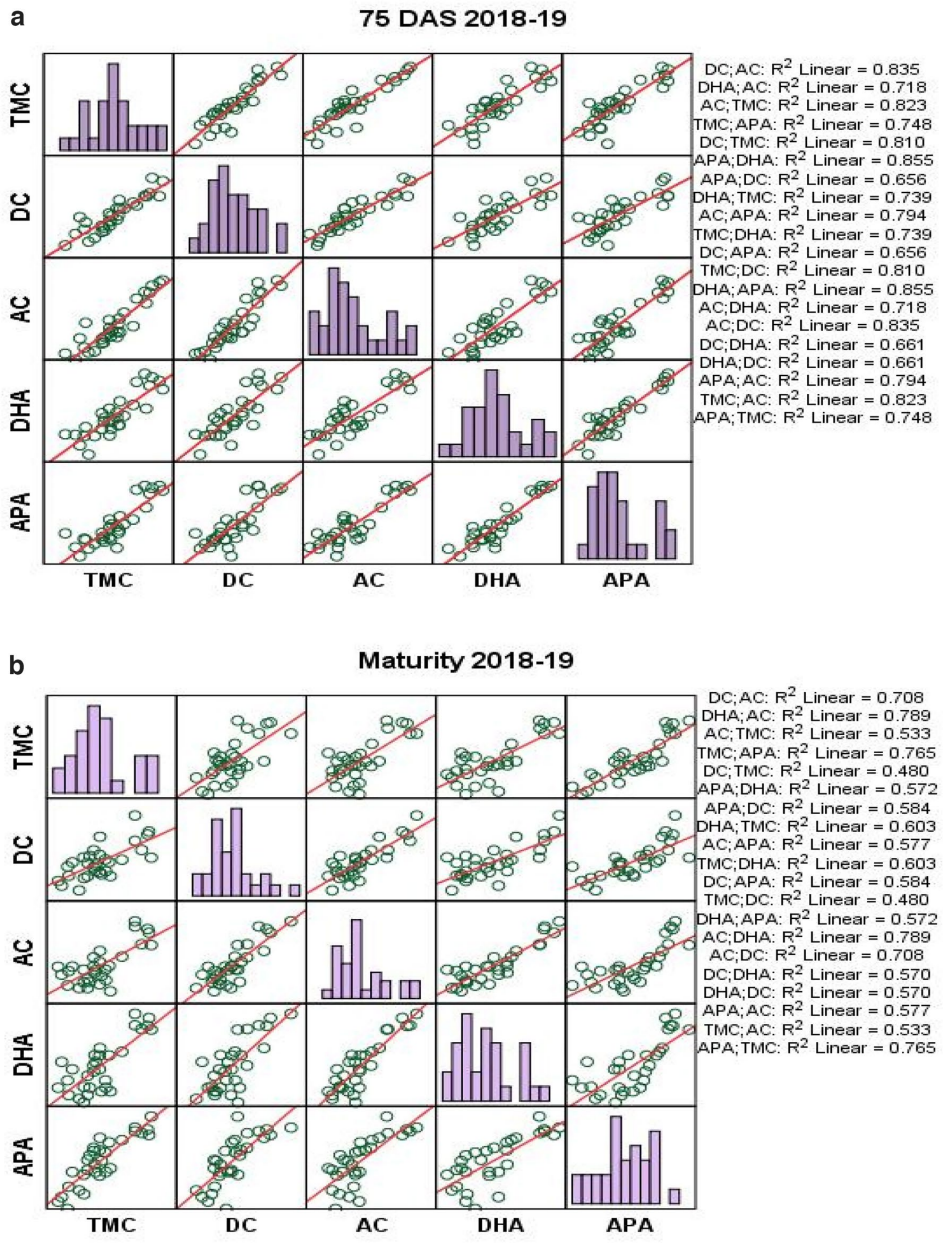

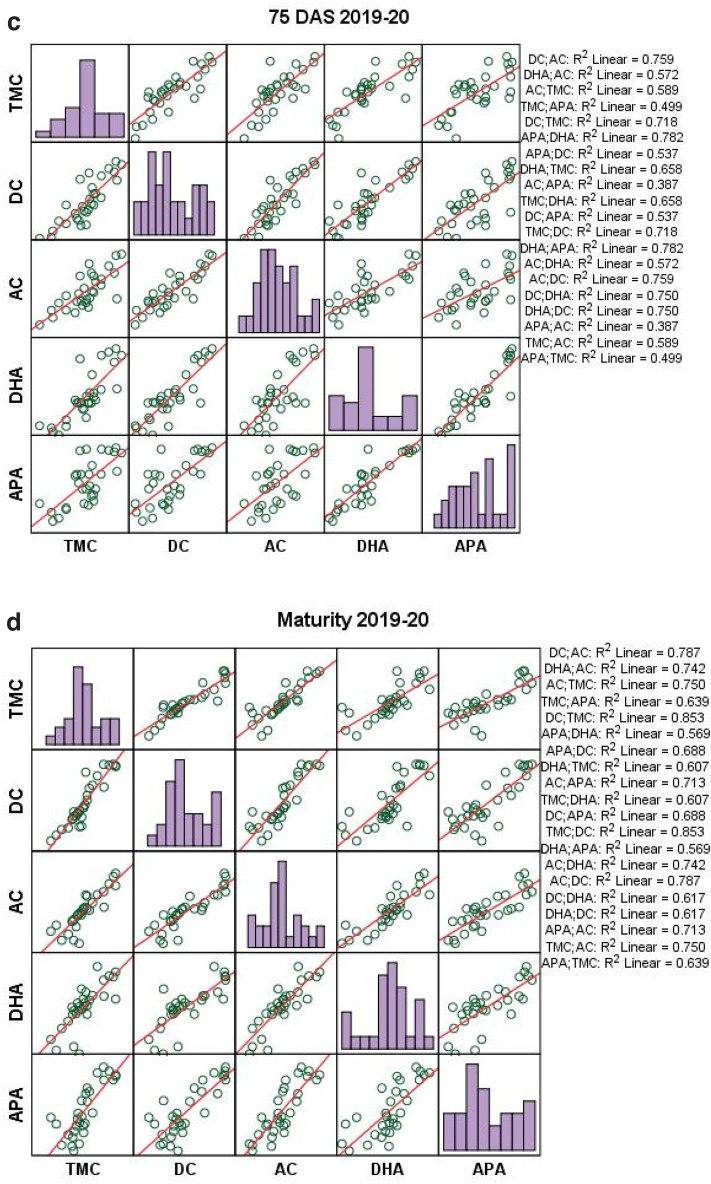
(**a−d**) Correlation analysis between microbial population and enzymatic activities at 75 DAS and at maturity during 2018–2019 and 2019–2020, respectively.

Rice residue management treatments showed significant correlation (p < 0.01 and p < 0.05) between microbial populations and enzymatic activity in wheat crop under rice–wheat cropping system in both cropping seasons (Fig. 7a–d). Total microbial count had strong positive correlation with dehydrogenase activity at 75 DAS (0.811–0.860) and at maturity (0.776–0.779), and alkaline phospatase activity at 75 DAS (0.706–0.865) and at maturity (0.799–0.875).

Diazotrophic count had significantly positive correlation with dehydrogenase activity at 75 DAS (0.813–0.866) and at maturity (0.755–0.785), and with alkaline phospatase activity at 75 DAS (0.733–0.810) and at maturity (0.764–0.830) (Fig. 7a–d). Similarly, actinomycetes activity had significantly positive correlation with dehydrogenase activity at 75 DAS (0.756–0.847) and at maturity (0.861–0.888), and alkaline phospatase activity at 75 DAS (0.622–0.891) and at maturity (0.760–0.845).

## Discussion

### Influence of rice residue management alternatives on soil microbial dynamics

Rice residue management in zero till wheat (ZTW) significantly influenced the total microbial count, diazotrophs, and actinomycetes. during the study seasons of 2018–2019 and 2019–2020. Soil microbial population gradually increased up to 75 DAS (anthesis stage) and decreased during later wheat growth period. In addition to production of noticeably higher SOC-up to 75 DAS, rice straw also had a greater impact on soil microbial composition (Figs. 5, 6). At crop maturity, however, this increase in the mineralization of the already-existing microbial community decreased. These dynamics most likely represent the residue's solubility or relative microbial accessibility in the field^44^. However, ZTW sown with Happy seeder enhanced soil microbial count by 23.7–38.3% and 47.9–60.4% and diazotropic count by 29.9–36.7% and 59.0–73.1% during 2018–2019 and 2019–2020, respectively, over residue burning and residue removal plots (Table 2). The sufficient amount of residue present in the soil, moderates soil temperature which helped in better multiplication of microbes during wheat anthesis and at maturity. Total microbial and diazotrophic count were higher than actinomycetes count under residue retained plots (ZTW using Happy Seeder, and ZTW without residue). It corroborated the findings of^38,45–47^. The populations of fungal, bacterial and actinomycetes were higher under ZT with surface residue retention than incorporation or removal in earlier studies as well^31,47^.

It is probable that these microbes have evolved to grow quickly in response to organic matter that is easily mineralized^48^. Concurrently CTW without residue (residue removal or burning) showed lower soil microbial count, diazotrophic count, and actinomycetes count compared to ZTW with partial or full residue retention. Immediately after burning of rice straw, top soil layer (0–3 cm) temperature is increased to 50–70 °C which affect drop down of heterotrophic microorganisms population from 77 to 9%^49^. Conventional tilled rice–wheat cropping without residue had the lowest microbial population^38^. According to Yadav et al.^50^, the rise in soil temperature during burning resulted in loss of actinomycetes population.

Despite the fact that our study focused on soil bacterial communities, soil fungi play an important role in SOC cycling^51^. Furthermore, soil fungi and their oxidative enzymes play a key role in the degradation of organic matter compounds with condensed aromatic ring structures, such as cellulose and lignin^52^. As a result, fungi are an excellent target for future research into the effects of rice residue on soil microbial communities.

### Influence of rice residue management alternatives on wheat productivity

During both study years, ZTW with Happy seeder in uniformly spread full residues produced significantly higher wheat yield (Fig. 2) as compared to CTW without residue (burning or removal) and CTW sown after chopper and spreader (drill sown/broadcast/spatial drill) (Figs. 2, 3). The wheat yield was enhanced by 9.8–11.3% during 2018–2019 and 2019–2020 under ZTW with Happy seeder (T7) over residue burning and residue removal plots. Increased crop residue decomposition likely increased nutrient availability, which in turn increased wheat yield by boosting the soil's microbial population and soil organic matter content^15,26,31,37,53,54^. Comparatively, higher wheat yield was recorded in ZTW drill sown after full residue removal and lowest was found in CTW broadcast sown with rotavator after using chopper and spreader with full residue load. ZT is the most important tillage method to conserve resources and enhance wheat yield^36,55,56^. Results from seeding wheat into rice residues using the Happy Seeder showed an increase in grain production by 9–15% over CTW in earlier studies^41,57,58^. Lowest yields under CTW with incorporation of full residues after chopper & spreader could be due to yellowing of plants during initial days because of N used by microbes for decomposition of incorporated residues.

### Influence of rice residue management alternatives on soil moisture content

During both the study years, ZTW sown with Happy seeder with full residue load exhibited higher soil moisture content compared to CTW with full residue incorporation and CTW without residue. ZT plays a significant role in improving soil moisture availability due to less soil compaction and uniform distribution of soil micro and macropores^15,26,31^. In contrast to tilled soil, un-tilled soil have less evaporative loss because ZTW with residue retention shields the soil surface from direct exposure to solar radiation and functions as a barrier to airflow over the soil surface. Similar results were reported by^38^, who found that ZT with residue had higher soil water content than ZTW without residue at two distinct depths (0–30 and 30–60 cm). Another study by^59^ found that no-till had an average soil water content that was between 13 and 14% greater than that of other tillage techniques.

### Influence of rice residue management alternatives on soil organic carbon

After a two-year crop cycle, ZTW with a partial or full residue load sown with a drill or Happy seeder significantly increased soil organic carbon in the upper 0–15 cm soil. Furthermore, soil organic carbon increased by 31% compared to CTW without residue or residue burning plots. Because of large-scale disruption of soil macro-aggregates under conventional tillage without residue and direct contact between microorganisms and straw, the retention of straw on the soil surface and subsequent ploughing increased C mineralization. Zero tillage and residue retention on the soil surface prevented direct microbial contact and provided the microbes with very few nutrients. As a result, ZT, in conjunction with residue retention, was found to be an important option for protecting the SOC and limiting C mineralization. Thus resulted in supply of more nutrients to the crop as well as enhanced water holding capacity and provided better aeration^18,53,60,61^. Due to the availability of a food source, crop residue retention promotes higher microbial population as compared to residue removal under ZT and CT^62,63^.

### Influence of rice residue management alternatives on soil enzymatic activity

Understanding soil microbial growth indices, fundamental biochemical processes, and fertility of any soil is greatly aided by the study of soil enzymatic activities^15,40^. However, during both study years, ZTW sown with Happy seeder with full residue and ZTW with Happy seeder sown after chopper and spreader with full residue load generated higher dehydrogenase and alkaline phosphatase activity as compared to CTW without residue (burning or removal) and CTW residue incorporation (Figs. 3, 4). Dehydrogenase activity was enhanced by 19.1–23.4% and alkaline phosphatase activity by 26.6–28.6%. Higher enzyme activities found in ZT plots could be due to two reasons.primarily, source of these enzymes are soil microbes, worms and insects, and microbial population is more in residue retained ZT wheat. Secondly, burning of the crop residue, may directly kill the microbes and a hydrolytic enzyme deactivated at high temperature during burning which may reduce the enzyme activity. Likewise, increased soil temperature indirectly altered dehydrogenase activity in soil^64^. According to Clarholm^65^, altering soil management procedures alters the microbial activity in the soil. Due to higher microbial activity in undisturbed soil and residue remaining on the soil surface, ZTW had higher dehydrogenase activity than CTW (because of active degradation of paddy straw). Similarly Gupta and Germida^66^, also observed increased dehydrogenase activity in conservation agriculture. However, in the current study, the lower alkaline phosphatase activity was found at harvest than at 75 DAS. This might be because of fewer substrates available to microbes and thus their population was less^67,68^. The extracellular production of the phosphatase enzyme is inhibited by an increase in soluble phosphorus^69^, and fertilisation with phosphatic fertiliser also suppresses phosphatase activity^70^. Residue retention enhances the nutrient mobilization and inhibits the fixation of available P by the soil. As a result, energy and a favorable environment for the accumulation of soil enzymes are provided. The current results are consistent with^66^ Gupta and Germida that the macro-aggregates had higher phosphatase activity in crop residues retention and ZT than CT in their respective micro-aggregates.

Soil enzymatic activity showed direct relationship with soil microbial population (Fig. 7a–d). Increased microbial populations like total microbial count, diazotrophic count and actinomycetes count increase the soil enzymatic activities like dehydrogenase and alkaline phosphatise activity. However, only actinomycetes and dehydrogenase activity had positive influence on grain and biomass yields of wheat. Residue management practices revealed a substantial and favourable association between enzyme activity and soil microbial count during the study period. Likewise^46^ reported that diazotrophic and actinomycetes count were positively correlated with dehydrogenase and alkaline phosphatise activity. These soil moicrobes are greatly enhanced under ZT conditions^26,38,71^.

### Influence of rice residue management alternatives on economics

During both the years, ZTW without full residue retention (after full residue burning, in anchored stubbles without or with partial burning) had the cost of cultivation lower than the ZTW sown with Happy seeder and other crop establishment methods after using chopper and spreader (drill sown, broadcast, spatial drill) and CTW without residue (burning or removal). Similarly, COC in ZTW Happy seeder sown wheat was comparatively higher than ZTW drill sown wheat but, I was lower than conventional tilled crop establishment methods and CTW residue removal. However, ZT drill and Happy seeder sown wheat yields were maximum than CTW residue incorporation and at par with CTW residue removal. Due this, net return and B-C ratio were higher. More number of field operations increased COC. Due to this reason different crop establishment methods under CT reduced B-C ratio. Sidhu et al^56^ reported that the cost of establishment with the Happy seeder was lesser than the establishment with a conventional method; nearly half of the expenditure of CT. The maximum gross return, net return and B-C ratio were found in wheat sown with zero tillage with residue retention followed by residue burned-zero tillage and residue removed-zero tillage^72,73^. As per^74^, Happy seeder zero tillage gave maximum net income (₹ 112,938 ha^−1^) with a B:C of 1:1.51 compared to conventional method with net income ₹ 102,602 ha^−1^ B:C of 1:1.33. Zero-till fertilizer-cum-seed-drill system was found as the most economical and gave the highest benefit–cost ratio than conventional wheat crop raising system and other reduced tillage systems^75^.

## Conclusion

The soil microbial characteristics of wheat were strongly impacted by various planting techniques and rice residue management techniques, and these consequently enhanced grain yield, soil organic carbon content and enzymatic activity. In ZTW seeded with Happy seeder under full surface residue retention (after chopper and spreader) enhanced soil microbial count and enzymatic activities by moderating the soil pH in the long run. Lowest soil microbial counts were observed under CTW after residue removal, showing a dynamic role of crop residues in promoting soil biological characteristics. Residue burning decreased the microbial populations, dehydrogenase and alkaline phosphatase activity. In rice–wheat cropping system, rice crop residues are needed to be managed by planting wheat preferably under ZT with residue retention using appropriate machine (like happy seeder) not only to sustain higher productivity and farm income but also to improve soil health and environmental quality.

Rice residue management in rice–wheat cropping system is an interdisciplinary endeavor combining technological innovations and sustainable agriculture practices with economic considerations and policy support. The agricultural sector can optimize residue management practices through these innovations for long-term productivity and environmental stewardship by addressing these aspects collaboratively in South Asia and at global level in the similar crop growing regions.

### Supplementary Information

Supplementary Tables.

## Data Availability

All data generated or analysed during this study are included in this published article and figure data are given in Supplementary file.
