# Supplementary material for: Rice residue management alternatives in rice–wheat cropping system: impact on wheat productivity, soil organic carbon, water and microbial dynamics
Source: Sci Rep. 2024 Jan 21;14:1822. doi: 10.1038/s41598-024-52319-6 (PMC10799959; doi:10.1038/s41598-024-52319-6)
Supplement: Supplementary file 1 — Supplementary Tables. [file 41598_2024_52319_MOESM1_ESM.docx]

Supplementary table 1: Grain and biological yields of wheat under variable rice residue management and crop establishment techniques during 2018-19 and 2019-20.

|  | Treatment | Grain yield (t ha^-1^) | | Biological yield (t ha^-1^) | |
| --- | --- | --- | --- | --- | --- |
|  |  | 2018-19 | 2019-20 | 2018-19 | 2019-20 |
| T1 | CTW drill sown after full residue burning | 6.02 | 6.14 | 13.06 | 13.21 |
| T2 | CTW drill sown after removal of residues (without burning) | 6.05 | 6.17 | 13.19 | 13.32 |
| T3 | ZTW with anchored stubbles | 5.93 | 6.03 | 12.89 | 13.05 |
| T4 | ZTW after anchored stubbles partial burning | 5.88 | 5.98 | 12.83 | 12.97 |
| T5 | ZTW after full residue burning | 5.86 | 5.96 | 12.78 | 12.91 |
| T6 | ZTW with Happy seeder in full residue load | 5.85 | 5.96 | 12.75 | 12.90 |
| T7 | ZTW with Happy seeder after using chopper & spreader (full residue load) | 5.66 | 5.68 | 12.44 | 12.53 |
| T8 | CTW broadcast sown with rotavator after using chopper & spreader (full residue load) | 5.58 | 5.61 | 12.18 | 12.32 |
| T9 | CTW drill sown after using chopper, spreader & rotavator (full residue load) | 5.60 | 5.65 | 12.25 | 12.38 |
| T10 | CTW with spatial drill after using chopper & spreader (full residue load) | 5.61 | 5.67 | 12.28 | 12.42 |
|  | SEm± | 0.09 | 0.09 | 0.13 | 0.11 |
|  | LSD (P≤0.05) | 0.27 | 0.27 | 0.38 | 0.34 |

Supplementary table 2: Dehdyrogenase activity and Alkaline phosphatase activity of wheat under variable rice residue management and crop establishment techniques during 2018-19 and 2019-20.

|  | Treatment | DHA µgTPF/g soil/24hr | | | | APA µgTPF/g soil/24hr | | | |
| --- | --- | --- | --- | --- | --- | --- | --- | --- | --- |
|  |  | 75 DAS | | Harvest | | 75 DAS | | Harvest | |
|  |  | 2018-19 | 2019-20 | 2018-19 | 2019-20 | 2018-19 | 2019-20 | 2018-19 | 2019-20 |
| T1 | CTW drill sown after full residue burning | 59.50 | 61.82 | 50.08 | 53.67 | 75.26 | 76.67 | 63.33 | 72.69 |
| T2 | CTW drill sown after removal of residues (without burning) | 63.12 | 63.25 | 51.33 | 54.00 | 77.82 | 77.50 | 63.40 | 75.82 |
| T3 | ZTW with anchored stubbles | 70.50 | 75.80 | 59.75 | 65.85 | 86.18 | 91.33 | 78.63 | 84.68 |
| T4 | ZTW after anchored stubbles partial burning | 68.50 | 74.40 | 55.58 | 64.90 | 83.21 | 89.80 | 75.88 | 82.03 |
| T5 | ZTW after full residue burning | 66.00 | 71.83 | 52.00 | 62.18 | 80.41 | 86.80 | 72.50 | 78.95 |
| T6 | ZTW with Happy seeder in full residue load | 75.75 | 86.05 | 67.21 | 70.03 | 93.75 | 97.65 | 80.50 | 87.33 |
| T7 | ZTW with Happy seeder after using chopper & spreader (full residue load) | 78.00 | 88.38 | 67.48 | 71.35 | 95.27 | 98.57 | 82.83 | 88.49 |
| T8 | CTW broadcast sown with rotavator after using chopper & spreader (full residue load) | 65.00 | 71.33 | 51.62 | 62.98 | 79.05 | 84.80 | 70.42 | 76.49 |
| T9 | CTW drill sown after using chopper, spreader & rotavator (full residue load) | 64.40 | 70.33 | 52.47 | 61.95 | 78.41 | 84.18 | 71.74 | 76.08 |
| T10 | CTW with spatial drill after using chopper & spreader (full residue load) | 65.42 | 70.08 | 52.41 | 63.93 | 78.41 | 85.00 | 71.42 | 75.51 |
|  | SEm± | 1.04 | 1.25 | 1.98 | 1.40 | 1.26 | 1.72 | 1.14 | 1.22 |
|  | LSD (P≤0.05) | 3.10 | 3.72 | 5.89 | 4.17 | 3.76 | 5.10 | 3.39 | 3.63 |

Supplementary table 3. Effect of rice residue management and wheat crop establishment methods on economics of wheat under rice-wheat cropping system (2018-19 and 2019-20)

| **S.N.** | **Treatment** | **Total cost**  **(x10^3^ ₹ ha^-1^)** | | **Gross return**  **(x10^3^ ₹ ha^-1^)** | | **Net return**  **(x10^3^ ₹ ha^-1^)** | | **B:C** | |
| --- | --- | --- | --- | --- | --- | --- | --- | --- | --- |
|  |  | **2018-19** | **2019-20** | **2018-19** | **2019-20** | **2018-19** | **2019-20** | **2018-19** | **2019-20** |
| T1 | **CTW drill sown after full residue burning** | 88.39 | 88.83 | 129.06 | 141.21 | 40.68 | 52.38 | 1.46 | 1.59 |
| T2 | **CTW drill sown after removal of residues (without burning)** | 88.79 | 89.29 | 129.83 | 141.97 | 41.04 | 52.68 | 1.46 | 1.59 |
| T3 | **Zero tillage wheat (ZTW) with anchored stubbles** | 81.49 | 81.93 | 127.22 | 139.02 | 45.73 | 57.08 | 1.56 | 1.70 |
| T4 | **ZTW after anchored stubbles partial burning** | 81.49 | 81.93 | 126.33 | 137.99 | 44.84 | 56.06 | 1.55 | 1.68 |
| T5 | **ZTW after full residue burning** | 81.49 | 81.93 | 125.88 | 137.48 | 44.39 | 55.54 | 1.54 | 1.68 |
| T6 | **ZTW with Happy seeder in full residue load** | 82.35 | 82.80 | 125.51 | 137.25 | 43.16 | 54.45 | 1.52 | 1.66 |
| T7 | **ZTW with Happy seeder after using chopper and spreader (full residue load)** | 83.79 | 84.23 | 122.02 | 131.95 | 38.24 | 47.71 | 1.46 | 1.57 |
| T8 | **CTW broadcast sown with rotavator after using chopper and spreader (full residue load)** | 89.82 | 90.27 | 119.97 | 130.08 | 30.15 | 39.81 | 1.34 | 1.44 |
| T9 | **CTW drill sown after using chopper, spreader and rotavator (full residue load)** | 91.26 | 91.70 | 120.44 | 130.89 | 29.17 | 39.19 | 1.32 | 1.43 |
| T10 | **CTW with spatial drill after using chopper and spreader (full residue load)** | 91.26 | 91.70 | 120.61 | 131.39 | 29.35 | 39.68 | 1.32 | 1.43 |
